# An injectable sulfonated reversible thermal gel for therapeutic angiogenesis to protect cardiac function after a myocardial infarction

**DOI:** 10.1186/s13036-019-0142-y

**Published:** 2019-01-17

**Authors:** David J. Lee, Maria A. Cavasin, Adam J. Rocker, Danielle E. Soranno, Xianzhong Meng, Robin Shandas, Daewon Park

**Affiliations:** 1 Department of Bioengineering, University of Colorado Denver Anschutz Medical Campus, Aurora, CO 80045 USA; 2 Department of Medicine, Division of Cardiology, University of Colorado Denver Anschutz Medical Campus, Aurora, CO 80045 USA; 3 Department of Pediatrics, University of Colorado Denver Anschutz Medical Campus, Aurora, CO 80045 USA; 4 Department of Surgery, University of Colorado Denver Anschutz Medical Campus, Aurora, CO 80045 USA

**Keywords:** Reversible thermal gel, Intramyocardial biomaterial injection, Therapeutic angiogenesis, Sulfonation, Heparin-mimicking, Spatiotemporal release

## Abstract

**Background:**

Cardiovascular disease and myocardial infarction are associated with high mortality and morbidity and a more effective treatment remains a major clinical need. The intramyocardial injection of biomaterials has been investigated as a potential treatment for heart failure by providing mechanical support to the myocardium and reducing stress on cardiomyocytes. Another treatment approach that has been explored is therapeutic angiogenesis that requires careful spatiotemporal control of angiogenic drug delivery. An injectable sulfonated reversible thermal gel composed of a polyurea conjugated with poly(N-isopropylacrylamide) and sulfonate groups has been developed for intramyocardial injection with angiogenic factors for the protection of cardiac function after a myocardial infarction.

**Results:**

The thermal gel allowed for the sustained, localized release of VEGF in vivo with intramyocardial injection after two weeks. A myocardial infarction reperfusion injury model was used to evaluate therapeutic benefits to cardiac function and vascularization. Echocardiography presented improved cardiac function, infarct size and ventricular wall thinning were reduced, and immunohistochemistry showed improved vascularization with thermal gel injections. The thermal gel alone showed cardioprotective and vascularization properties, and slightly improved further with the additional delivery of VEGF. An inflammatory response evaluation demonstrated the infiltration of macrophages due to the myocardial infarction was more significant compared to the foreign body inflammatory response to the thermal gel. Detecting DNA fragments of apoptotic cells also demonstrated potential anti-apoptotic effects of the thermal gel.

**Conclusion:**

The intramyocardial injection of the sulfonated reversible thermal gel has cardioprotective and vascularization properties for the treatment of myocardial infarction.

**Electronic supplementary material:**

The online version of this article (10.1186/s13036-019-0142-y) contains supplementary material, which is available to authorized users.

## Background

Atherosclerosis is a progressive disease that can lead to coronary heart disease and myocardial infarction if atherosclerotic plaque accumulates in coronary arteries. Coronary heart disease is prevalent among 6.3% of the United States adult population and is the underlying cause of one in every seven deaths, while myocardial infarction is prevalent in 7.9 million adults and occurs approximately every 40 s [1]. Following myocardial infarction, the higher stress induced on the surviving cardiomyocytes results in pathological cardiac remodeling involving ventricular dilation and ventricular wall thinning that ultimately advances to heart failure.

Cardiac function after myocardial infarction can be improved through the injection of biomaterials into the ventricular wall of an infarcted heart and reducing left ventricular wall stress by mechanical load shielding, increasing ventricular wall thickness, and decreasing ventricle radius [2]. Intramyocardial injections can be utilized to increase capillary density and vascularization, reduce cardiac hypertrophy and fibrosis, increase cardiac progenitor cell recruitment, and reduce cardiomyocyte apoptosis [3–5]. Biomaterials may also serve as drug delivery systems for the spatiotemporal release of angiogenic factors to overcome the issues of short protein half-lives and rapid diffusion from target sites [6]. The inclusion of angiogenic factors to biomaterials may further improve cardiac function in intramyocardial injection therapy. Biomaterial injections combined with the controlled release of biologicals may encourage cardiac regeneration and functional improvement as the material will localize and sustain biomolecule delivery, while protecting the biologic factors and extend their half-live in vivo [7]. The use of angiogenic factors as therapeutic proteins to treat myocardial ischemia aims to increase the perfusion to the surviving cardiomyocytes and preserve cardiac function. The angiogenic process is initiated by the binding of angiogenic factors to endothelial cell receptors that trigger the formation of new vessels that eventually mature by stabilization with perivascular cells [8]. VEGF is a predominant growth factor involved in mediating angiogenesis [9]. Clinical trials involving angiogenic factors have demonstrated the need for prolonged tissue exposure for the development of robust and sustained vascularization, and required for the survival of newly formed vasculature [10]. Due to the rapid diffusion, poor stability, and shot half-lives of angiogenic factors, supraphysiological doses or multiple injections are needed, which leads to excessive uncontrolled vascular formation in undesired locations resulting in unstable vessel growth that resembles immature tumor vasculature [11]. The electrostatic interaction between heparin sulfate and angiogenic factors allows for drug binding, stabilization of receptors, and protection from proteolysis, and biomaterials functionalized with heparin have been shown to exhibit sustained delivery of angiogenic factors [12]. Electrostatic and biochemical interactions utilize electrostatic or biochemical affinity between the biomaterial and biological factors to control release. The incorporation of heparin in a hydrogel system can be used to regulate drug release, while maintaining bioactivity and improving controlled microvessel growth [13].

An injectable sulfonated reversible thermal gel composed of poly(serinol hexamethylene urea) (PSHU) conjugated with poly(N-isopropylacrylamide) (PNIPAM) and sulfonate groups (SPSHU-PNIPAM) has been developed for therapeutic angiogenesis [14, 15]. The PSHU backbone has been shown to exhibit therapeutic effects in neuronal [16–18] and cardiac [14, 19] tissue engineering applications. Temperature responsive biomaterials utilize their hydrophobic and hydrophilic components to physically respond at different temperatures. These biomaterials are soluble below their lower critical solution temperature (LCST), but as temperature is increased, they become increasingly hydrophobic and insoluble, leading to hydrogel formation. PNIPAM has a LCST of 32 °C and has been used to provide temperature responsive properties for the controlled release of drugs [20], and may be injected clinically in a minimally invasive manner with a robotic injection system incorporating a temperature-controlled injectate line for intramyocardial injection therapy [21]. The release of positively charged angiogenic factors may be prolonged by exploiting heparin-binding interactions through sulfonate groups. The focus of this study was to evaluate the sulfonated reversible thermal gel for the protection of cardiac function after a myocardial infarction.

## Methods

### Materials

Serinol, urea, hexamethylene diisocyanate (HDI), N,N-dimethylformamide (DMF), 4,4′-azobis(4-cyanovaleric acid), 1,3-propanesultone, and Masson trichrome stain kit were purchased from Sigma-Aldrich (St. Louis, MO, USA). Di-tert-butyl dicarbonate, ethyl acetate, trifluoroacetic acid (TFA), 2,2,2-trifluoroethanol (TFE), N-(3-dimethylamino- propyl)-N′-ethylcarbodiimide hydrochloride (EDC), N-hydroxysuccinimide (NHS), potassium tert-butoxide, and dimethyl sulfoxide (DMSO) were purchased from Alfa Aesar (Ward Hill, MA, USA). Hexane and diethyl ether were purchased from Fisher Scientific (Pittsburgh, PA, USA). N-isopropylacrylamide (NIPAM) was purchased from Tokyo Chemical Industry (Tokyo, Japan). Methylene chloride (DCM) was purchased from JT Baker (Phillipsburg, NJ, USA). IRDye 800CW NHS ester was purchased from LI-COR (Lincoln, NE, USA). Recombinant murine VEGF165 was purchased from PeproTech (Rocky Hill, NJ, USA). Cluster of differentiation 31 (CD31) primary antibody (rat IgG2a), alpha smooth muscle actin (SMA) primary antibody (rabbit IgG), Alexa Fluor 488 secondary antibody (goat anti-rat IgG), Alexa Fluor 594 secondary antibody (goat anti-rabbit IgG, rabbit anti-goat IgG) were purchased from Thermo Fisher Scientific (Waltham, MA, USA). von Willebrand factor (VWF) primary antibody (sheep IgG) and cluster of differentiation 68 (CD68) primary antibody (rabbit IgG) were purchased from Abcam (Cambridge, United Kingdom). 4′,6-diamidino-2-phenylindole (DAPI) Fluoromount-G was purchased form Electron Microscopy Sciences (Hatfield, PA, USA). CardioTACS In Situ Apoptosis Detection Kit was purchased from Research and Diagnostic Systems (Minneapolis, MN, USA).

### Equipment

Fluorescent optical images were taken using the LI-COR Pearl Impulse. Confocal images were taken using the Zeiss LSM 780. Echocardiographic images were obtained using FUJIFILM VisualSonics Vevo 2100 equipped with a 30 MHz cardiac scanhead transducer. Brightfield images were obtained using the Nikon Eclipse Ti-E.

### Synthesis of sulfonated reversible thermal gel: SPSHU-PNIPAM

N-BOC serinol was synthesized as described previously [18]. Briefly, serinol and di-tert-butyl dicarbonate were dissolved in ethanol at 4 °C. The solution was heated at 37 °C for 1 h, rotoevaporated, and dissolved in an equal volume mixture of ethyl acetate and hexane at 60 °C. Additional hexane was added to form crystalline structures and the precipitate was filtered to remove solvent, yielding N-BOC serinol as a crystalline white product.

PNIPAM was synthesized as described previously [22]. In short, NIPAM and 4,4′-azobis(4-cyanovaleric acid) were dissolved in methanol and heated at 68 °C for 3 h. PNIPAM was recovered by precipitation in ultrapure water at 60 °C, purified via dialysis (molecular weight cutoff (MWCO) 12–14 kDa), and lyophilized, yielding a white product.

SPSHU-PNIPAM was synthesized similarly as described previously [14]. N-BOC serinol, urea, and HDI were dissolved in DMF and heated at 90 °C for 7 days. PSHU was recovered by precipitation in diethyl ether and rotoevaporation, yielding the polyurea as a white powder. PSHU was dissolved in DCM and TFA. Deprotection occurred by hydrogenation at room temperature for 45 min providing free amine groups. Deprotected PSHU (dPSHU) was recovered by precipitation in diethyl ether and rotoevaporation. Further purification of dPSHU involved dissolving in TFE, precipitation in diethyl ether, and rotoevaporation. Next, an equivalent mass of PNIPAM was conjugated to dPSHU. PNIPAM, EDC, and NHS were dissolved in DMF and activated for 24 h. Separately, dPSHU was dissolved in DMF, added to the activated PNIPAM solution, and reacted for 24 h. PSHU-PNIPAM was recovered by precipitation in diethyl ether, rotoevaporation, purified via dialysis (MWCO 12–14 kDa), and lyophilized. Afterwards, the remaining free amine groups were sulfonated. 1,3-propanesultone, potassium tert-butoxide, and PSHU-PNIPAM were dissolved in DMF and reacted at 60 °C for 3 days. SPSHU-PNIPAM was recovered by precipitation in diethyl ether, rotoevaporation, purified via dialysis (MWCO 12–14 kDa), and lyophilized, yielding a light-yellow product. Polymer characterization was performed as described previously to confirm material produced [15, 17].

### Angiogenic growth factor release test

IRDye 800CW NHS ester (0.15 mg/ml; 99% PBS, 1% DMSO) was reacted with VEGF (0.1 mg/ml, PBS) at a 0.03 dye to growth factor mass ratio at pH 8.5 for 2 h. A desalting column was used to remove excess dye to purify the labeled VEGF solution.

Animal procedures involved in the in vivo dye-labeled VEGF release test study were approved by the Institutional Animal Care and Use Committee (IACUC). C57BL/6 J mice (The Jackson Laboratory) weighing 24–28 g were maintained on a light/dark (14 h light, 10 h dark) cycle with access to food and water ad libitum. The study involved 3–4 mice per injection group (VEGF, PSHU-PNIPAM + VEGF, SPSHU-PNIPAM + VEGF) for five time points (0, 1, 3, 7, 14 days). The mice were anaesthetized using continuous isoflurane and medical air inhalation. Initial induction was at 5% isoflurane and then maintained at 2% isoflurane. The rodents were continuously monitored on a heated platform. A 5 mm median cervical skin incision was made, and the lobes of the thyroid gland and their isthmus were separated to expose the sternohyoideus muscle and trachea. The inner needle of a trocar was removed and gently inserted inside the trachea serving as an intubation tube. Artificial ventilation was provided at 2% isoflurane, tidal volume at 150–260 μl/stroke, and ventilation rate at 130–160 strokes/min. An oblique incision 10 mm long at a site 2 mm away from the left sternal border in the direction of where the left front leg meets the body was made and the thoracic muscle cut to expose the ribs. The chest cavity was opened with a 6–8 mm incision in the third intercostal space with chest retractors used to gently pull back and open the incision. The pericardium was gently pulled apart. Either VEGF (1000 ng, 30 μl saline), PSHU-PNIPAM + VEGF (1%, 500 ng VEGF, 30 μl saline), or SPSHU-PNIPAM + VEGF (1%, 500 ng VEGF, 30 μl saline) was injected intramyocardially at three areas with equal volume at the injury site through a 31 gauge needle. The hydrogels gelled instantly upon injection. The chest cavity was closed by suturing the incision in the third intercostal space followed by the suturing of muscle and skin layers.

The mice were euthanized by carbon dioxide and cervical dislocation after 0, 1, 3, 7, or 14 days of injection. The skin, muscle, and sternum around the chest were removed and the heart exposed before fluorescent optical images were taken (Additional file 1: Figure S1). Florescence intensities were converted to VEGF mass by a standard curve generated by measuring intensities of known VEGF standard amounts (Additional file 1: Figure S2).

### Myocardial infarction reperfusion injury mouse model

Animal procedures involved in the myocardial infarction reperfusion injury and injection study were approved by the IACUC. C57BL/6 J mice (The Jackson Laboratory) weighing 24–28 g were maintained on a light/dark (14 h light, 10 h dark) cycle with access to food and water ad libitum. The study involved 7–12 mice per treatment group (saline, VEGF, SPSHU-PNIPAM, SPSHU-PNIPAM + VEGF, no injection) that survived 28 days after myocardial infarction reperfusion injury. The mice were anaesthetized using continuous isoflurane and medical air inhalation. Initial induction was at 5% isoflurane and then maintained at 2% isoflurane. The rodents were continuously monitored on a heated platform. A 5 mm median cervical skin incision was made, and the lobes of the thyroid gland and their isthmus were separated to expose the sternohyoideus muscle and trachea. The inner needle of a trocar was removed and gently inserted inside the trachea serving as an intubation tube. Artificial ventilation was provided at 2% isoflurane, tidal volume at 150–260 μl/stroke, and ventilation rate at 130–160 strokes/min. An oblique incision 10 mm long at a site 2 mm away from the left sternal border in the direction of where the left front leg meets the body was made and the thoracic muscle cut to expose the ribs. The chest cavity was opened with a 6–8 mm incision in the third intercostal space with chest retractors used to gently pull back and open the incision. The pericardium was gently pulled apart. The left anterior descending coronary artery was ligated along with a piece of PE-10 tubbing using 8–0 suture. The wound was temporarily closed ribs and skin were closed using 4–0 suture. After 45 min of ischemia, the heart was exposed again and reperfusion of the myocardium started by removing the suture around the PE-10 tubing. Either saline (30 μl saline), VEGF (500 ng, 30 μl saline), SPSHU-PNIPAM (1%, 30 μl saline), or SPSHU-PNIPAM + VEGF (1%, 500 ng VEGF, 30 μl saline) was injected intramyocardially at three areas with equal volume at the injury site through a 31 gauge needle or no injection was administered for the associated group. The hydrogels gelled instantly upon injection. The chest cavity was closed by suturing the incision in the third intercostal space followed by the suturing of muscle and skin layers.

### Evaluating cardiac function with echocardiography

Serial transthoracic echocardiography was performed while simultaneously recording ECG to assess cardiac morphology and left ventricular function. The mice were anaesthetized using continuous isoflurane and medical air inhalation. Initial induction was at 5% isoflurane and then maintained at 1.5% isoflurane at 1.5 l/min. Long and short parasternal axes views of left ventricle were obtained with the mice maintained on a heated platform at 37 °C. Short axis two-dimensional views of the left ventricle at the papillary muscle level were used to obtain M-mode targeted recordings. All measurements were averaged from three consecutive cardiac cycles on the exhale phase.

### Cardiac tissue harvest

Cardiac tissue was harvested 28 days after myocardial infarction and injection. The mice were anaesthetized using continuous isoflurane and oxygen inhalation. Initial induction was at 5% isoflurane in oxygen and then maintained at 2% isoflurane in oxygen. The thoracic cavity was opened and while the heart still beating, potassium chloride (10%, saline) was injected through the posterior basal region and into the left ventricular chamber through a 31 gauge needle to arrest the heart in diastole, after which the heart was harvested. The cardiac tissue was embedded in OCT and frozen at − 80 °C. The cardiac tissue was sectioned transversely starting at the apex with a thickness of 5 μm.

### Trichrome staining of cardiac tissue

The sections were washed in running deionized water to remove OCT compound and fixed in Bouin solution (71% saturated aqueous picric acid, 24% formaldehyde, 5% acetic acid) at 56 °C for 15 min, cooled in deionized water at room temperature, and washed in running deionized water. Biebrich scarlet/acid fuchsin solution (0.9% Biebrich scarlet, 0.1% acid fuchsin, 1.0% acetic acid) was used for 5 min and the sections washed in running deionized water. The sections were placed in a phosphotungstic/phosphomolybdic acid solution (2.5% phosphotungstic acid, 2.5% phosphomolybdic acid) for 5 min. Aniline blue solution (2.4% Aniline blue, 2% acetic acid) was used for 5 min. The sections were placed in 1% acetic acid for 2 min and washed in running deionized water. Subsequent washes consisted of 70% ethanol for 1 min and 100% ethanol for 1 min. The sections were cleared in xylene for 2 min and mounted. Infarct size was measured from brightfield images of five trichrome stained cardiac tissue sections (960 μm, 1440 μm, 1920 μm, 2400 μm, and 2880 μm away from the apex) and averaged.

### Immunohistochemistry

The sections were fixed in formalin (10%, PBS) for 10 min and washed three times with wash buffer (0.1% Tween 20, PBS) for 5 min each. Permeabilization buffer (0.5% Triton X-100, PBS) was used for 10 min and the sections washed three times with wash buffer for 5 min each. Blocking buffer (0.25% Triton X-100, 2% BSA, 4% bovine gamma globulins, PBS) was used for 60 min on the sections at room temperature. All antibodies were diluted in dilution buffer (0.25% Triton X-100, 2% BSA, 4% bovine gamma globulins, PBS). The sections were stained with primary antibodies CD31 (1:50), VWF (1:50), SMA (1:250), and/or CD68 (1:500) overnight at 4 °C and washed three times in wash buffer for 5 min each. The sections were stained with secondary antibodies Alexa Fluor 488 (1:500) for CD31 and/or the associated Alexa Flour 594 (1:500) for VWF, SMA, and CD68 at room temperature. The sections were washed three times in wash buffer for 5 min each and washed three times in ultrapure water for 5 min each. DAPI Fluoromount-G was used to stain nuclei and mount the sections. Cell and vessel counts from confocal images of the immunohistochemically stained cardiac tissue were obtained for three to five random visual fields for sections 1920 μm from the apex in the infarct area or ischemic border zone with z-stack projections (4 μm thickness, 1 μm steps) and averaged. Stains associated with DAPI were used to designate cells positive for a specific stain.

### TUNEL assay of cardiac tissue

The CardioTACS In Situ Apoptosis Detection Kit was used to detect DNA fragmentation generated from apoptosis. The sections were fixed in formalin (10%, PBS) and washed three times with PBS for 5 min each. Proteinase K solution (1:200) was used for 15 min and the sections washed two times in deionized water for two min each. The sections were immersed in quenching solution (90% methanol, 3% hydrogen peroxide, 7% deionized water) for 5 min and washed in deionized water for 1 min. TdT labeling buffer was used for 5 min on the sections. Labeling reaction mix (1% TdT dNTP mix, 1% 50X manganese (2+), 1% TdT enzyme, 97% TdT labeling buffer) used for 60 min at 37 °C on the sections before immersing sections in TdT stop buffer for 5 min and washed two times in deionized water for 5 min each. Strep-HRP solution (0.125% Strep-HRP, 99.875% blue Strep-HRP diluent) (1:800) was used for 10 min and washed in deionized water for 5 min each. The sections were incubated with TACS-Blue Label for 2 min and washed two times in deionized water for 2 min each. The sections were immersed in Nuclear Fast Red for 2 min and washed three times in deionized water for 2 min each. Subsequent wash consisted of 95% ethanol for 1 min. The sections were cleared in xylene for 2 min and mounted. Cell counts from brightfield images were obtained for four to five random visual fields for sections 1920 μm from the apex in the ischemic border zone and averaged.

### Statistical analysis

Two-tailed t-test assuming unequal variances was used to determine significant differences between two groups. Analysis of variance (ANOVA) was used to determine significant differences between three or more groups followed by Tukey-Kramer to determine significant differences between two groups as appropriate. Statistical significance was considered when *p* < 0.05.

## Results and discussion

### SPSHU-PNIPAM reduced the initial burst release of VEGF compared to PSHU-PNIPAM and showed continued release after 14 days

**Fig. 1:**
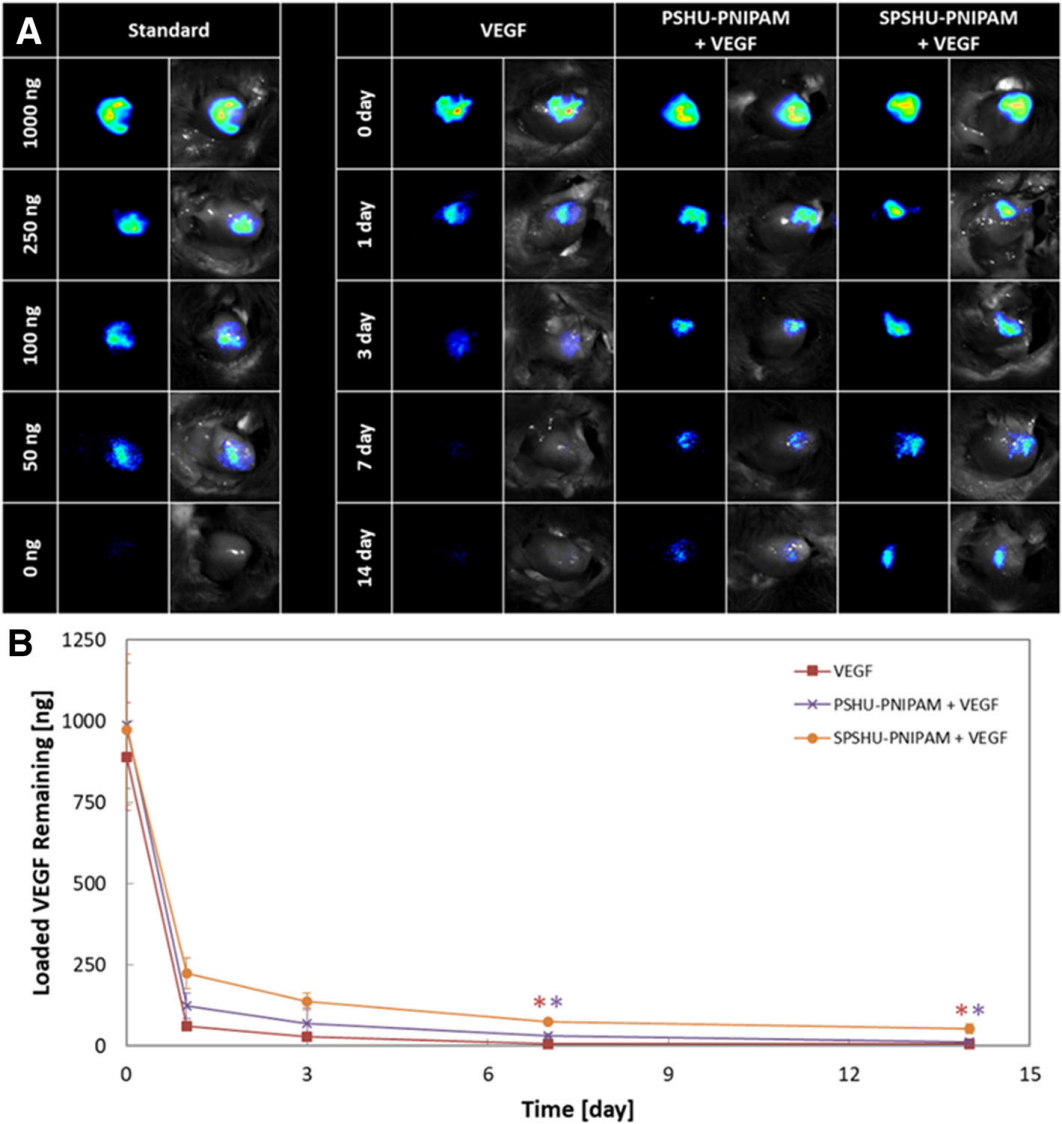
In vivo VEGF release profile after intramyocardial injection in left ventricular wall. **a** fluorescent optical images showing localized VEGF relative to known amounts of VEGF as standards, **b** VEGF remaining in the heart. *n* = 3, error bars represent standard deviation, and * indicates *p* < 0.05

An in vitro angiogenic growth factor release test for the release of VEGF has been previously investigated, but primarily accounts for diffusion [14]. The in vivo release test was designed to account for release mechanisms by oxidative and enzymatic degradation as well as diffusion to represent the release of VEGF under actual physiological conditions. The release characteristics of VEGF loaded in hydrogels after an intramyocardial injection was examined (Fig. 1).

The optimal angiogenic sprouting occurs when the release profile of VEGF consists of a high initial concentration followed by a decreasing concentration over time [23]. Compared to a bolus injection of VEGF, the VEGF loaded in hydrogels presented more sustained release. All groups showed a significant burst release and rapid clearance from the heart within the first day. The large burst release of VEGF from the hydrogels may be due to the limited loading capabilities of the hydrogels. Within 7 days, the bolus injection of VEGF was completely cleared from the heart as no measurable amount of VEGF was detected. PSHU-PNIPAM slowed the clearance of VEGF, but no significant amount of VEGF was remaining after 14 days. However, the sulfonation groups in SPSHU-PNIPAM provided a decrease in the initial burst release compared to PSHU-PNIPAM and localized VEGF can still be seen after 14 days. The formation of stable vessels requires VEGF expression or release for over two weeks as short term VEGF release leads to unstable vessel formation and vessel regression after the cessation of angiogenic stimulus [24, 25]. The results suggest that the SPSHU-PNIPAM hydrogel can decrease the rate of which VEGF is cleared from the myocardium, allowing for an increase in localized VEGF concentration that may improve angiogenic response in the heart.

### Cardiac function improved with SPSHU-PNIPAM thermal gel injection

**Fig. 2:**
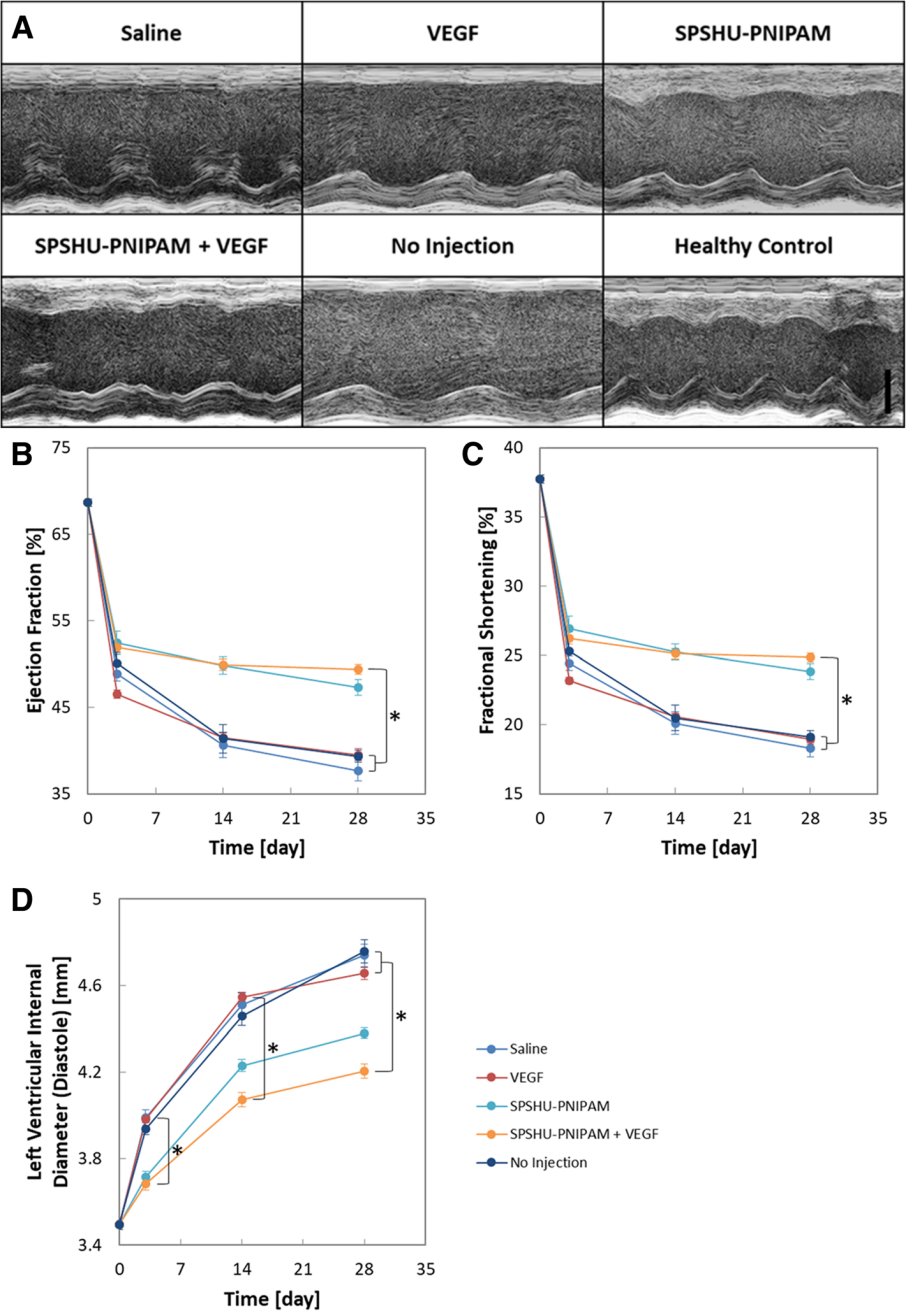
Assessment of cardiac function after myocardial infarction reperfusion injury treated with intramyocardial injection. **a** M-mode echocardiogram after 28 days. Scale bar represents 2 mm. Echocardiography measurements of cardiac function **b** ejection fraction, **c** fractional -shortening, **d** left ventricular internal diameter at diastole. *n* = 7–12, error bars represent standard error of the mean and * indicates *p* < 0.05

After myocardial infarction, cardiac function decreases and can be evaluated with ejection fraction, fractional shortening, and left ventricle inner diameter that can be measured using echocardiography [26]. Ejection fraction is the fraction of blood contained in the ventricle at the end of diastole that is expelled during its contraction and decreases as cardiac function decreases. Fractional shortening is the reduction of the length of the end-diastolic diameter that occurs by the end of systole and decreases as cardiac function decreases. Left ventricle inner diameter increases as cardiac function decreases. These measurements obtained with echocardiography was used to evaluate cardiac function after myocardial infarction reperfusion injury treated with intramyocardial injection (Fig. 2).

Although the decrease in cardiac function due to the myocardial infarction reperfusion injury was observed, the improvements of ejection fraction, fractional shortening, and left ventricular internal diameter were used to evaluate treatment effects. Ejection fraction improved for the hydrogel groups compared to the saline, VEGF, and no injection controls. Intramyocardial injections of SPSHU-PNIPAM either loaded with or without VEGF seemed to have very similar treatment effects for ejection fraction and fractional shortening. The loading of VEGF in SPSHU-PNIPAM did not significantly improve cardiac function and indicates that the more significant component of the cardioprotective effects of the hydrogel system is the biomaterial itself rather than the release of VEGF. The use of SPSHU-PNIPAM may allow for augmentation of left ventricular wall thickness and resulting in reconstruction of left ventricular geometry and improvement of cardiac function [27]. However, the only statistically significant improvement that was observed over the saline, VEGF, and no injection controls was SPSHU-PNIPAM + VEGF. This may demonstrate the combined effects of the individual improvements to cardiac function from vascularization via sustained VEGF release as well as the favorable properties of the SPSHU-PNIPAM biomaterial. Additional cardioprotective effects of sustained VEGF release may be magnified by optimizing VEGF release characteristics including loading amount and release rate. Investigating cardiac function after 28 days, allowing more time for nascent vessels to mature and revascularize the infarcted cardiac tissue may also provide more insight to the effects of therapeutic angiogenesis.

### SPSHU-PNIPAM thermal gel injection reduced infarct size and ventricular wall thinning

**Fig. 3:**
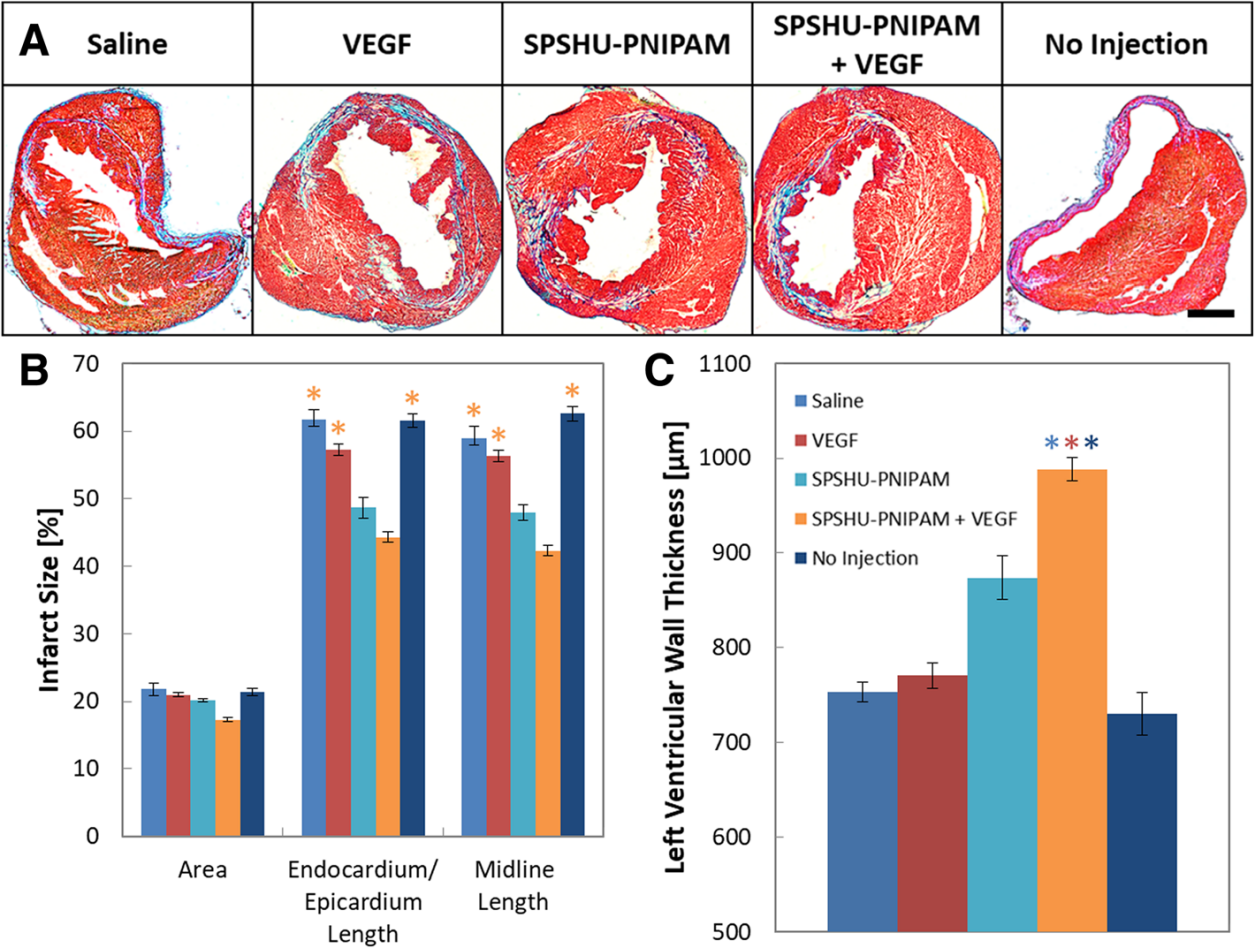
Determining extent of infarct 28 days after myocardial infarction reperfusion injury treated with intramyocardial injection. **a** Masson trichrome images 1920 μm from the apex. Muscle fibers (red), cytoplasm (pink), and collagen from fibrotic tissue (blue). Scale bar represents 1000 μm. Quantification of **b** infarct size and **c** left ventricular wall thickness. n = 7–12, error bars represent standard error of the mean and * indicates *p* < 0.05

The extent of the infarct size after myocardial infarction reperfusion injury, treated with a intramyocardial injection, can be used to evaluate treatment effect. Masson trichrome stain allows for differentiation of healthy myocardium in red and pink from the infarct areas that are characteristic of fibrotic or collagenous tissue formation in blue (Fig. 3).

The severity of the myocardial injury was assessed by infarct size and wall thinning. Infarct size can be evaluated using serval techniques, including by area, endocardium and epicardium length, and midline length [28]. Infarct size based on area was calculated by dividing the sum of infarct areas from the total area of the left ventricle. Infarct size based on endocardium and epicardium length was calculated as the average infarct length of the endocardium and epicardium. Infarct length was taken as the ratio of the length of circumferential infarct surface that included greater than 50% of the whole thickness of myocardium to the total circumferential length. Infarct area based on midline length was calculated as the ratio of the midline circumferential infarct length that included greater than 50% of the whole thickness of the myocardial wall to the total circumferential midline length. These different methods were used to determine infarct size and evaluate the treatment effects on reducing myocardial injury. Another approach to measure the extent of the infarct that ensues myocardial infarction reperfusion injury is the left ventricular wall thickness. Due to the loss of cardiomyocytes from ischemic damage, higher stress on the surviving cardiomyocytes leads to left ventricular dilation and thinning of the ventricular wall, and a thicker wall would indicate a less substantial infarct.

The approach based on area to measure infarct size resulted in values with low differences between treatment groups, however, length based approaches were more sensitive as greater differences were observed for each injection group. SPSHU-PNIPAM + VEGF demonstrated the lowest infarct size after myocardial infarction reperfusion injury and was statistically reduced compared to the saline, VEGF, and no injection controls. The decrease in infarction size shows that the reduced injury in the myocardium, after a myocardial infarction, could potentially increase the survival of cardiomyocytes that will improve cardiac function and limit cardiac remodeling. Similarly, left ventricular wall thickness showed that the SPSHU-PNIPAM + VEGF treatment group reduced the wall thinning resulting from myocardial infarction. An increasing treatment effect was observed with a thicker ventricular wall for the SPSHU-PNIPAM group as well and may be attributed to the increased mechanical stability with the intramyocardial injection of the biomaterial.

### SPSHU-PNIPAM thermal gel injection increased functional vascular endothelial cell count, vessel count, and vessel stabilization by perivascular cells

**Fig. 4:**
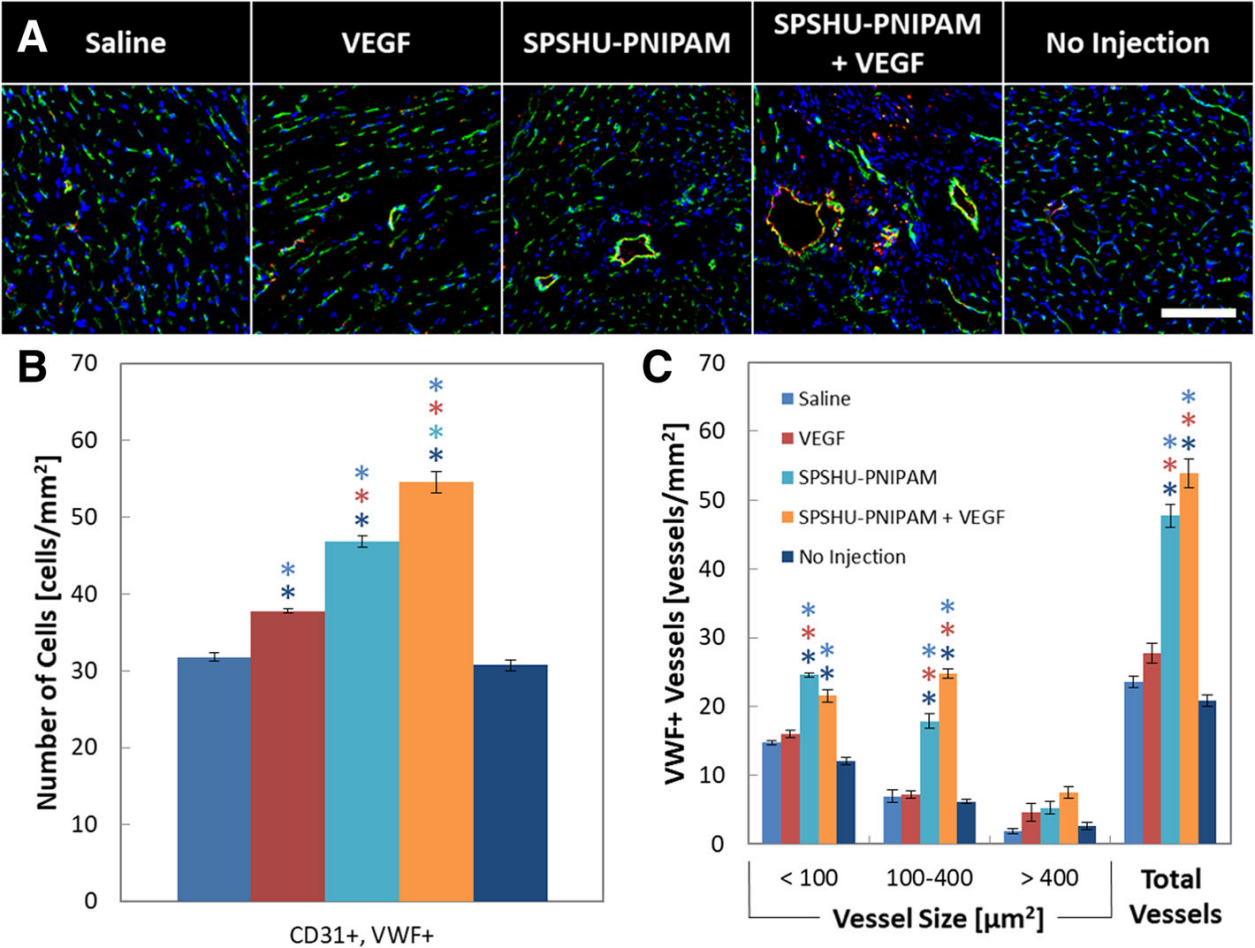
Immunohistochemical assessment of vascularization by vessel formation, and functional vascular endothelial cell count 28 days after myocardial infarction reperfusion injury. **a** images of CD31 and Alexa Fluor 488 (green), VWF and Alexa Fluor 594 (red), and DAPI (blue). Functional vascular cells were characteristic of CD31+ and VWF+. Scale bar represents 100 μm. Quantification of immunohistochemical assessment of vascularization by **b** functional vascular endothelial cell counts and **c** vessel counts with functional vascular endothelial cells. *n* = 7–12, error bars represent standard error of the mean and * indicates *p* < 0.05

The angiogenic process is initiated by the binding of angiogenic factors to endothelial cell receptors that trigger the formation of new vessels that eventually mature by stabilization with perivascular cells [8]. Immunohistochemistry was used to evaluate the vascularization effects of the intramyocardial thermal gel injections loaded with VEGF. CD31 was used to observe the presence of endothelial cells and VWF for endothelial cells that have differentiated into functional vascular endothelial cells (Fig. 4).

The immunohistochemical results for vascularization show that an increase in functional vascular endothelial cells and vessel formation result after hydrogel injections. The larger vessels that formed may be attributed to the high concentrations of localized VEGF [29]. The hydrogel groups showed an increase in the number of functional vascular endothelial cells compared to the saline, VEGF, and no injection controls, and vessels positive for functional vascular endothelial cells show an increased number of vessels. Although there may have been a minimal increase in functional vascular endothelial cell count and vessel formation with SPSHU-PNIPAM + VEGF, there was no significant difference compared to SPSHU-PNIPAM. Intramyocardial injection of hydrogels has been shown to induce vascularization even without the incorporation of angiogenic factors [30]. The promotion of angiogenesis may be attributed to inflammation induced vascularization, including VEGF dependent vascularization that is initiated via signal transducer and activator of transcription 3 (STAT3) pathway that is induced by the pro-inflammatory cytokine interleukin 6 (IL-6) [14, 31, 32].

**Fig. 5:**
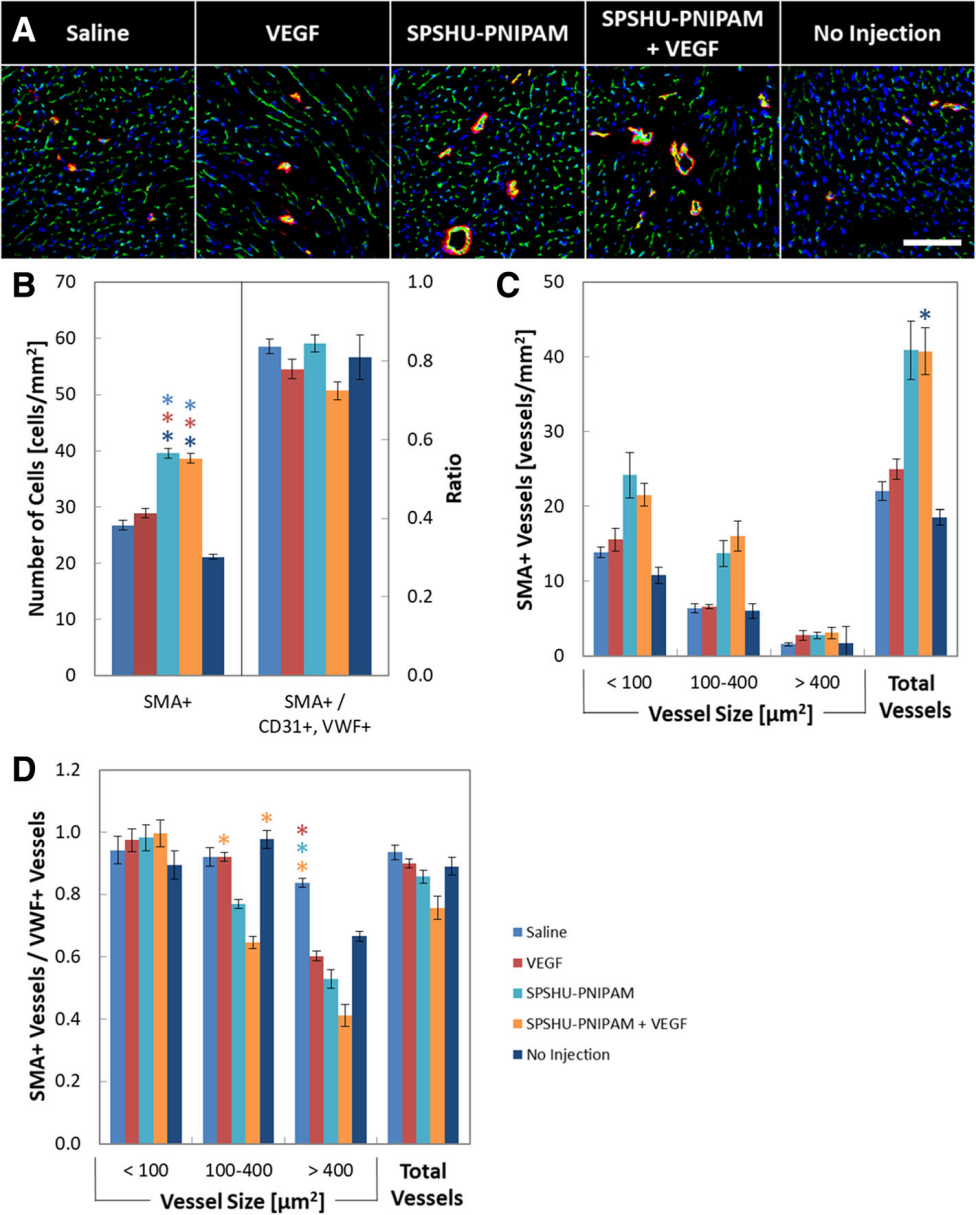
Immunohistochemical assessment of vascularization by vessel formation, functional vascular endothelial cell count, and vascular smooth muscle cell count 28 days after myocardial infarction reperfusion injury. **a** images of CD31 and Alexa Fluor 488 (green), SMA and Alexa Fluor 594 (red), and DAPI (blue). Vascular smooth muscle cells were characteristic of SMA+ associated with CD31+. Scale bar represents 100 μm. Quantification of immunohistochemical assessment of vascularization by **b** vascular smooth muscle cell counts and ratio of vascular smooth muscle cells to functional vascular endothelial cells, **c** vessel counts with vascular smooth muscle cells, and **d** ratio of vessels with vascular smooth muscle cells to vessels with functional vascular endothelial cells. *n* = 7–12, error bars represent standard error of the mean and * indicates *p* < 0.05

Although intramyocardial thermal gels were demonstrated to induce vessel formation, the maturation of these vessels needs to be assessed to determine their functionality as blood vessels. SMA was used to observe the presence of vascular smooth muscle cells and evaluate the stabilization of vessels (Fig. 5).

Consistent with the increase in functional vascular endothelial cells after hydrogel injections, vascular smooth muscle cell counts and the number of vessels with vascular smooth muscle cells increased. However, there was a lack in vessel maturation of larger vessels. When comparing the maturation of the vessels shown by the ratio of SMA+ vessels to VWF+ vessels, no difference was observed for small vessels. However, maturation of larger vessels for the hydrogel injections decreased compared to the saline, VEGF, and no injection controls. Improved vascularization and perfusion of the cardiac tissue through larger vessels may be vital to reducing myocardial infarction injury and may explain the insignificant increase in cardiac function with VEGF release compared to thermal gel alone. Although the sustained release of VEGF improved the maturation of smaller vessels, the maturation of larger vessels observed with the promotion of late stage angiogenesis through additional angiogenic factors (e.g. platelet-derived growth factor (PDGF)) may be required for functional larger vessels [33].

### Inflammatory response to SPSHU-PNIPAM thermal gel injection was limited

**Fig. 6:**
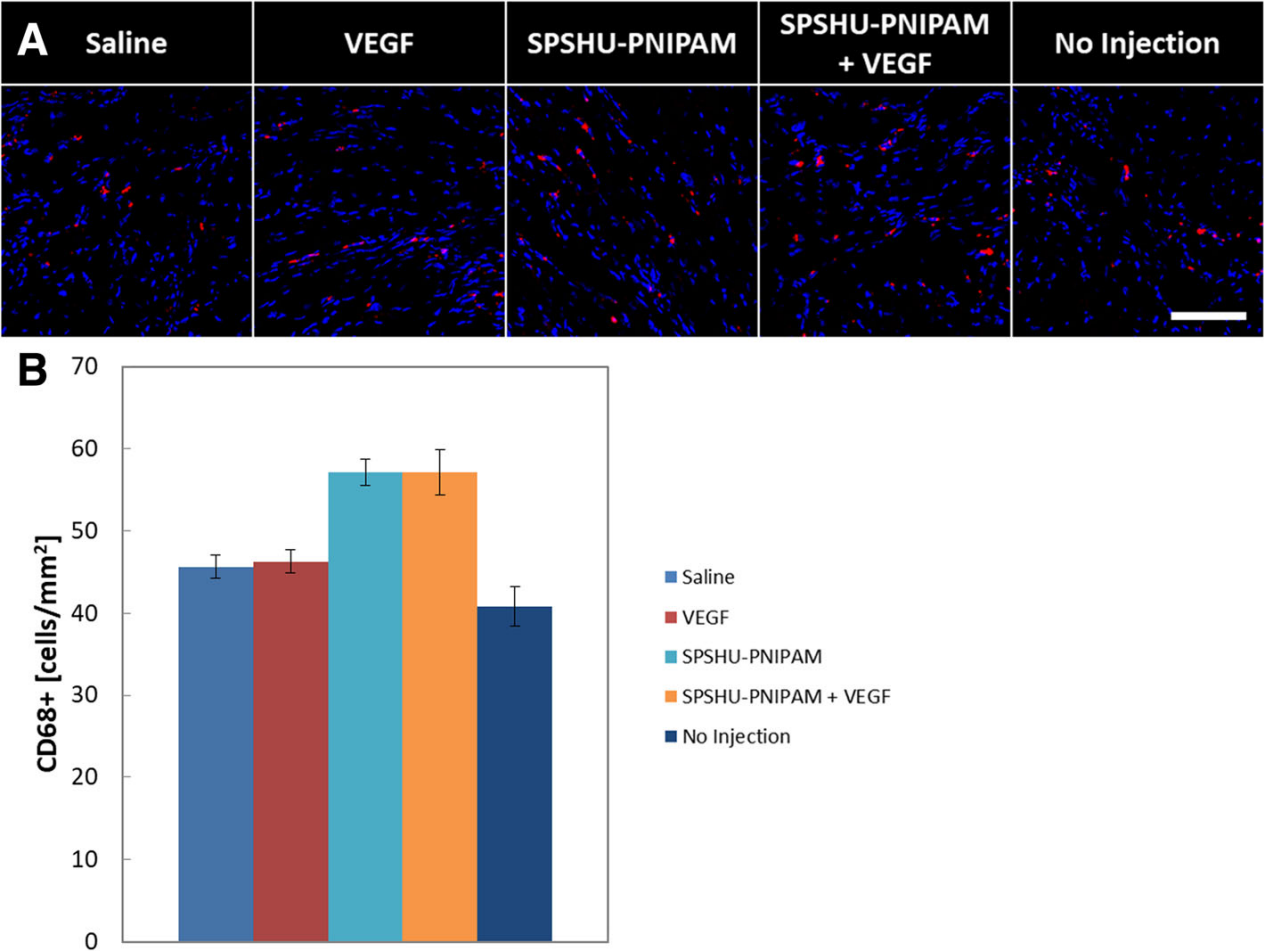
Immunohistochemical assessment of inflammatory response by macrophage cell count 28 days after myocardial infarction reperfusion injury. **a** CD68 and Alexa Fluor 594 (red) and DAPI (blue). Scale bar represents 100 μm. **b** quantification of immunohistochemical assessment of inflammatory response by macrophage cell count. *n* = 7–12, error bars represent standard error of the mean

In order to assess the inflammatory response to the hydrogel injections relative to the response seen with myocardial infarction, immunohistochemistry was used (Fig. 6).

After the initial infiltration of macrophages following myocardial infarction, macrophages begin to clear the infarct area and there is more inflammation in the remote regions of the infarct by 28 days that is due to a secondary inflammatory response that occurs during cardiac remodeling [34]. Assessing inflammation 28 days after myocardial infarction will allow for the assessment of inflammation due to a foreign body relative to the inflammation due to the myocardial infarction. A slight increase in the number of macrophages was observed for the hydrogel injections but was not statistically significant. These results indicate the inflammatory response due to the ischemic injury is predominate compared to the inflammatory response due to the foreign body injection.

### SPSHU-PNIPAM thermal gel injection reduced number of apoptotic cells

**Fig. 7:**
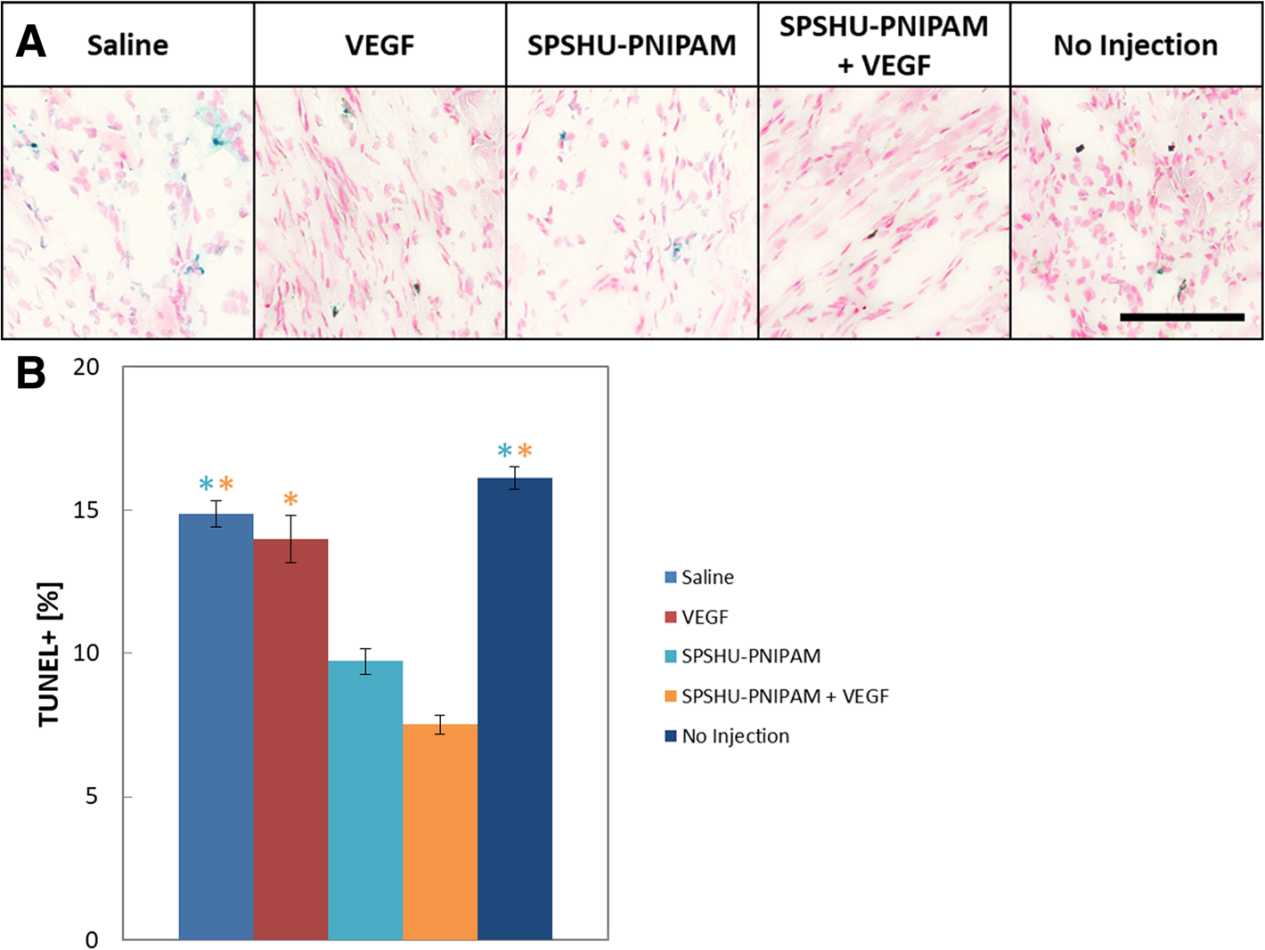
Apoptosis assessment 28 days after myocardial infarction reperfusion injury. **a** TUNEL assay 1920 μm from the apex. Nuclei of non-apoptotic cells (red) and nuclei of apoptotic cells (blue), scale bar represents 100 μm. **b** quantification of apoptotic cells. *n* = 7–12, error bars represent standard error of the mean and * indicates *p* < 0.05

Cell survival was another method to determine the treatment effect of the different groups. Following myocardial infarction reperfusion injury, cardiomyocytes become highly apoptotic to the ischemic environment. By decreasing the number of apoptotic cardiomyocytes, cardiac function will improve after injury. A TUNEL assay was used to measure the number of apoptotic cells (Fig. 7).

Apoptosis is a form of cell death that is induced by ischemia reperfusion injury that can be detected by labeling the ends of DNA strand breaks that are specific to the apoptotic process [35]. After 28 days of ischemic injury, the number of apoptotic cells is significantly reduced for the hydrogel injections compared to the saline, VEGF, and no injection controls. However, there are several limitations of this assessment. The TUNEL stain does not distinguish between cardiomyocytes and other cells at the site of injury [36]. Also, this assessment shows apoptotic cells after 28 days of injury, while a better timepoint for this assessment may be within a few days of injury since the majority of cardiomyocyte death occurs over the first 6–24 h of ischemia with an increasing lesser amount of cell death for months [37].

## Conclusions

The sulfonated reverse thermal gel, SPSHU-PNIPAM, loaded with VEGF was evaluated for therapeutic angiogenesis to protect cardiac function after myocardial infarction. The electrostatic binding affinity of SPSHU-PNIPAM to VEGF, from the addition of sulfonate groups, allowed for sustained, localized delivery of VEGF for two weeks, which potentially formed stable blood vessels. The protection of cardiac function and vascularization of infarcted myocardium after intramyocardial thermal gel injection in a myocardial infarction reperfusion injury mouse model was evaluated. Treatment with SPSHU-PNIPAM consistently showed improved cardiac function and vascularization, but the additional delivery of VEGF showed very limited additional therapeutic benefits. Further investigation should include optimizing VEGF release characteristics including both loading amount and release rate. The decline of ejection fraction and fractional shortening after myocardial infarction were reduced, while left ventricular internal diameter showed reduced ventricular dilation. Both infarct size and left ventricular wall thinning decreased while an increase in the vessel formation was observed. These results demonstrate the biomaterial SPSHU-PNIPAM, has cardioprotective and increased vascularization properties for the treatment of myocardial infarction.

## Additional file

Additional file 1: **Figure S1.** Representative fluorescent optical images showing localized VEGF after exposing heart. **Figure S2.** Standard curve of intensities of fluorescent optical images with corresponding VEGF standard amounts. Error bars represent standard deviation. (DOCX 891 kb)
