# Supplementary material for: An injectable sulfonated reversible thermal gel for therapeutic angiogenesis to protect cardiac function after a myocardial infarction
Source: J Biol Eng. 2019 Jan 17;13:6. doi: 10.1186/s13036-019-0142-y (PMC6337754; doi:10.1186/s13036-019-0142-y)
Supplement: Supplementary file 1 — Figure S1. Representative fluorescent optical images showing localized VEGF after exposing heart. Figure S2. Standard curve of intensities of fluorescent optical images with corresponding VEGF standard amounts. Error bars represent standard deviation. (DOCX 891 kb) [file 13036_2019_142_MOESM1_ESM.docx]

**An injectable sulfonated reversible thermal gel for therapeutic angiogenesis to protect cardiac function after a myocardial infarction**

David J. Lee ^1^, Maria A. Cavasin ^2^, Adam J. Rocker ^1^, Danielle E. Soranno ^1,3^, Xianzhong Meng ^4^, Robin Shandas ^1^, Daewon Park ^1,*^

^1^ Department of Bioengineering, University of Colorado Denver Anschutz Medical Campus, Aurora, CO 80045 USA

^2^ Department of Medicine, Division of Cardiology, University of Colorado Denver Anschutz Medical Campus, Aurora, CO 80045 USA

^3^ Department of Pediatrics, University of Colorado Denver Anschutz Medical Campus, Aurora, CO 80045 USA

^4^ Department of Surgery, University of Colorado Denver Anschutz Medical Campus, Aurora, CO 80045 USA

Corresponding Author

*Email: daewon.park@ucdenver.edu


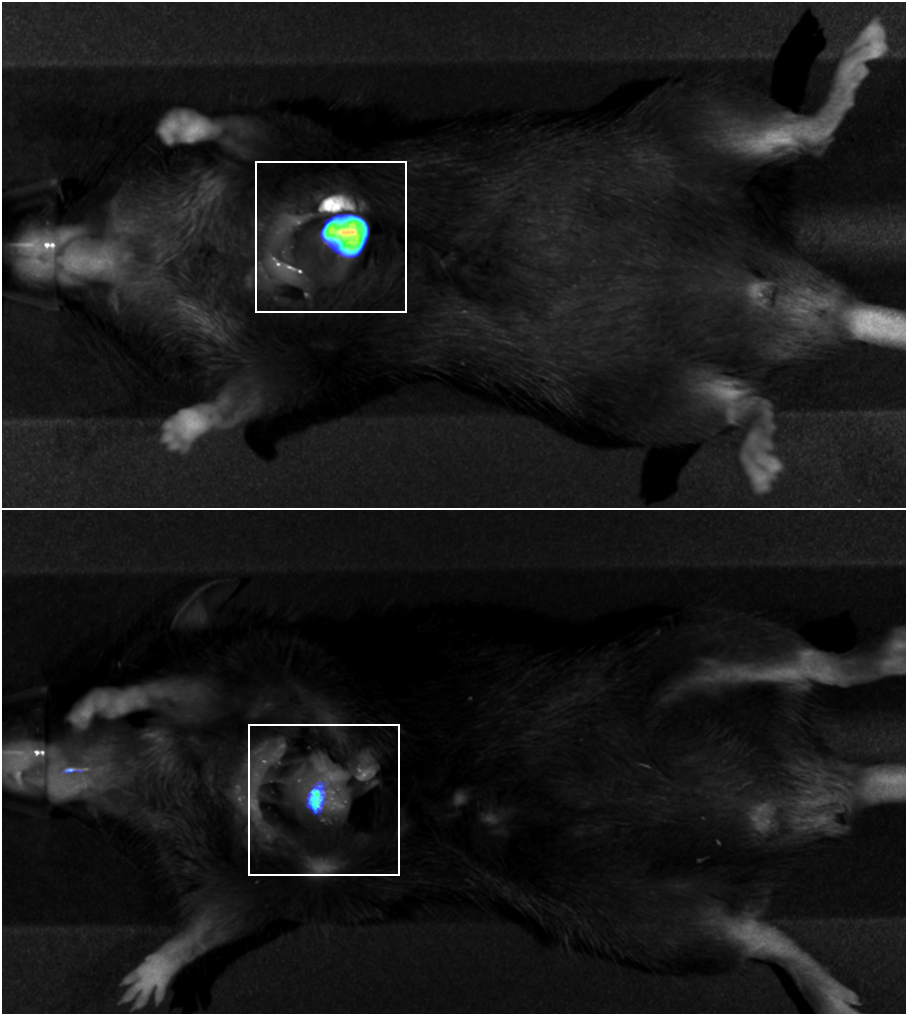


**Figure S1.** Representative fluorescent optical images showing localized VEGF after exposing heart.


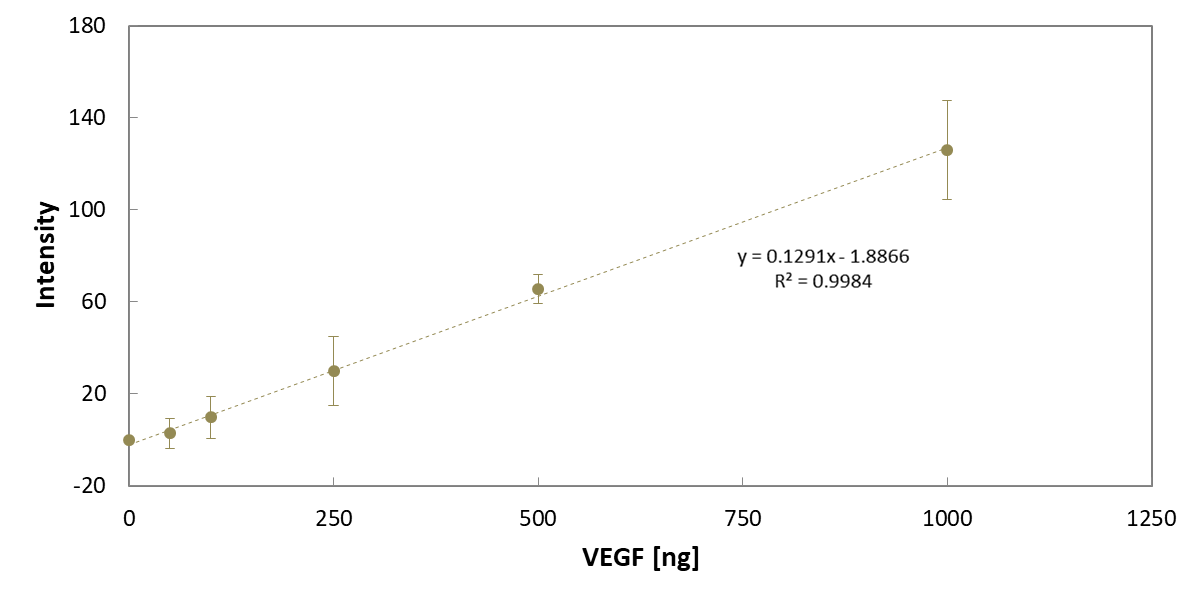


**Figure S2.** Standard curve of intensities of fluorescent optical images with corresponding VEGF standard amounts. Error bars represent standard deviation.
